# In Vivo Modeling of Human Breast Cancer Using Cell Line and Patient-Derived Xenografts

**DOI:** 10.1007/s10911-022-09520-y

**Published:** 2022-06-13

**Authors:** Eric P. Souto, Lacey E. Dobrolecki, Hugo Villanueva, Andrew G. Sikora, Michael T. Lewis

**Affiliations:** 1 Lester and Sue Smith Breast Center, Baylor College of Medicine, Houston, TX 77030 USA; 2 Otolaryngology-Head and Neck Surgery, Baylor College of Medicine, Houston, TX 77030 USA; 3 Department of Head and Neck Surgery, Division of Surgery, University of Texas MD Anderson Cancer Center, Houston, TX 77030 USA; 4 Departments of Molecular and Cellular Biology and Radiology, Baylor College of Medicine, Houston, TX 77030 USA; 5 Dan L Duncan Comprehensive Cancer Center, Baylor College of Medicine, Houston, TX 77030 USA; 6 Baylor College of Medicine, One Baylor Plaza, BCM-600; Room N1210, Houston, TX 77030 USA

**Keywords:** In vivo models, Patient-derived xenograft, Immunodeficient mouse and rat models, Preclinical drug screening, Preclinical trials

## Abstract

Historically, human breast cancer has been modeled largely in vitro using long-established cell lines primarily in two-dimensional culture, but also in three-dimensional cultures of varying cellular and molecular complexities. A subset of cell line models has also been used in vivo as cell line-derived xenografts (CDX). While outstanding for conducting detailed molecular analysis of regulatory mechanisms that may function in vivo, results of drug response studies using long-established cell lines have largely failed to translate clinically. In an attempt to address this shortcoming, many laboratories have succeeded in developing clinically annotated patient-derived xenograft (PDX) models of human cancers, including breast, in a variety of host systems. While immunocompromised mice are the predominant host, the immunocompromised rat and pig, zebrafish, as well as the chicken egg chorioallantoic membrane (CAM) have also emerged as potential host platforms to help address perceived shortcomings of immunocompromised mice. With any modeling platform, the two main issues to be resolved are criteria for “credentialing” the models as valid models to represent human cancer, and utility with respect to the ability to generate clinically relevant translational research data. Such data are beginning to emerge, particularly with the activities of PDX consortia such as the NCI PDXNet Program, EuroPDX, and the International Breast Cancer Consortium, as well as a host of pharmaceutical companies and contract research organizations (CRO). This review focuses primarily on these important aspects of PDX-related research, with a focus on breast cancer.

## Introduction

In recent years, several laboratories worldwide have developed comparatively large collections of patient-derived xenograft (PDX) models of human cancers in highly immunocompromised mouse hosts. PDX establishment in mice has been particularly effective for breast cancer [1–12]. More recently, other modeling systems such as, rat, pig and zebrafish have also been exploited as hosts for in vivo growth and analysis of human tumors. In addition, the chicken egg chorioallantoic membrane (CAM) experimental model has been adapted from its over 100-year role as a tool for developmental biology studies to attempt growth of a variety of cancers, including breast [13].

In all hosts, and for all cancers, credentialing PDXs as clinically-relevant models remains a major stumbling block, primarily due to the time and money it takes to characterize PDXs fully, preferably with respect to the tumors-of-origin. Several mouse PDX collections are far along in this regard, using benchmarked annotation and omic analysis pipelines [14–17]. With formation of international PDX consortia, such as the International Breast Cancer PDX Consortium [7], PDXNet of the National Cancer Institute (USA) (www.pdxnetwork.org), and EuroPDX (EU) (www.europdx.eu), and with the activities of the Patient-derived Models Repository (PDMR) of the National Cancer Institute (NCI) (www.pdmr.cancer.gov), as well as with significant contributions from commercial enterprises including pharmaceutical companies and contract research organizations (Table 1), the community has made rapid progress in generating and credentialing mouse PDX models of a number of cancers [7, 14, 16, 17].

**Table 1:** Representative List of CROs That Provide PDX and Drug Screening Services

**Company**	**Homepage**
Aveo Oncology	https://www.aveooncology.com
Bioduro-Sundia	https://bioduro-sundia.com
BioReperia	https://bioreperia.com/pdx-models/
Certis Oncology	https://www.certisoncology.com/#drug-development
Champions Oncology	https://www.championsoncology.com
Charles River Laboratories	https://www.criver.com
Crownbio	https://www.crownbio.com
Envigo	https://www.envigo.com
Hera BioLabs	https://www.herabiolabs.com
The Jackson Laboratory	https://www.jax.org
Oncodesign	https://www.oncodesign.com
StemMed	https://stemmedcancer.com
Taconic	https://www.taconic.com
WuXI AppTec	https://us.wuxiapptec.com
Xenograft	https://www.xenograft.net

Specifically with respect to breast cancer PDX credentialing in mice, the community has shown recapitulation of the following relative to the tumor of origin (with a few exceptions): 1) clinically-relevant biomarker expression, 2) histological features, 3) cellular heterogeneity, 4) cell division rates, 5) patterns of metastasis, 6) variant allele frequencies, 7) genomic copy number alterations, 8) mRNA gene expression patterns, 9) protein expression patterns, and 10) general concordance of treatment response with their respective tumors-of-origin [3, 7, 14, 16–21]. While clonal heterogeneity can show drift [14], the community has shown that, in general, PDX models are stable over multiple transplant generations at the genomic, transcriptomic, and proteomic levels, thereby offering confidence that experimental results should be repeatable with serial passaging in vivo [3, 7, 14, 16, 17]. Similar data have been published for several other organ sites [22–28]. In aggregate, these data also provide a certain level of confidence that results from PDX-based studies can be translated into the clinic effectively.

While PDX-based experiments have already provided a wealth of information, particularly in the evaluation of candidate therapeutics, there are limitations to these studies. The primary limitations are time and money to conduct large preclinical trials of 20 + PDX lines. Further, individual PDX collections, generally speaking, are not yet large enough to reflect the full spectrum of human disease. In fact, many tumor types are difficult to grow in immunocompromised mice (e.g. ER + and HER2 + breast cancer, though the high frequency of ER + breast cancers compensates for their poor PDX establishment rate, and alternative methods have improved engraftment rates [29]), or are rare in the population (e.g. angiosarcoma of the breast, phyllodes tumors) and are thus not readily available for engraftment. The lack of the immune system in immunocompromised hosts precludes some investigations entirely, or renders others more difficult to conduct and analyze (e.g. studies involving humanization of the mouse immune system). Additionally, lack of access to clinical samples or animal facilities, even aside from budgetary limitations, can make PDX research impractical, or impossible, for some research groups to conduct. To overcome these limitations, at least in part, new host species may need to be engineered, and new techniques must be developed to exploit PDX-derived tissue more effectively.

Also remaining to be developed are more refined protocols for evaluating drug responses that mimic the clinical setting as closely as possible, and a more granular assessment of the degree to which PDX drug responses match those of the tumor-of-origin. In order to accomplish this, pharmacokinetic (PK) and pharmacodynamic (PD) differences between mouse and human for each agent under study, as well as the route of administration, must be considered. If these limitations can be overcome, PDX models stand an excellent chance of accelerating translational research, and making a significant impact on patient outcomes and survival, to a greater degree than they do currently.

## Contributions of PDX-Based Research on Clinical Research and Trials

Cell line xenograft and PDX-based research has contributed greatly to clinical trial design and translational research [30]. In fact, virtually every therapeutic clinical trial conducted in the last two to three decades was based, in whole or in part, on results derived from either cell line xenografts or PDX, or both. Table 2 shows selected examples of PDX-based studies that were conducted either at BCM or collaborating institutions that were subsequently validated clinically, or are currently being evaluated in clinical trials. Similar tables could be built for all institutions conducting therapeutic clinical trials. As more of these trials begin reporting, the impact of PDX-based research will become clear, and that impact is expected to be substantial.

**Table 2:** Selected Clinical Trials Based On PDX Studies Conducted At BCM or Collaborating Institutions

**Target**	**Agent**	**Company**	**PDX-based Rationale and Clinical Impact**	**Reference(s)**
Gamma Secretase	MK-0752	Merck & Co	Drug sequencing in PDX determined the sequencing in the clinical trial Trial: NCT00645333	[208]
Receptor Tyrosine Kinases (Multiple)	Crizotinib + Sunitinib (C + S)	Pfizer	In 14 PDX, C + S showed efficacy, particularly in models with low PTPN12 expression, led to a C + S clinical trial, and subsequent shift to Sitravatinib, which has the same target range as C + S as a single agent. The clinical trial is being designed currently Trial: CRIZENT—NCT02074878	[187]
Chemotherapy Combinations	Docetaxel + Carboplatin	Docetaxel – Sanofi-Aventis Carboplatin – Bristol-Myers Squibb	In PDX, combination showed a higher response rate than either single agent. The result was additive for those responsive to docetaxel and those responsive to carboplatin. Only ~ 10% of PDX showed enhanced response over the best single agent, with ~ 10 showing a worse response to combination vs. the best single agent. Clinically, the combination also showed a higher response rate than historical single agent efficacy Trial: CADENCE-NCT02547987 Trial: NCT02124902	[19]
STAT3	TTI-101	Tvardi Therapeutics	In vivo results in breast cancer xenografts indicated efficacy, in combination with docetaxel for TNBC. The clinical trial includes breast cancer and is ongoing Trial: NCT03195699	[209, 210]
Microtubule Polymerization and PARP1/2	Eribulin + Talazoparib	Eribulin – Eisai Co Talazoparib – Pfizer	PDX-derived organoids showed response that was confirmed in a patient-matched PDX. The patient had a recurrence and was treated successfully with the combination Trial: N/A	[12]
ERN1 (IRE1)	ORIN 1001	Fosun Pharma	PDX chosen on the basis of MYC expression showed that high MYC PDX responded to ERN1 inhibition. The clinical trial is ongoing Trial: NCT03950570	(188)

## In Vivo Modeling Platforms

Preclinical animal models have been essential to biomedical research for decades. They have enabled researchers to elucidate the pathological mechanisms driving cancer and preclinically evaluate new drugs. Genetically engineered mouse models (GEMMs) and mouse-derived immunocompetent allograft models have also contributed significantly to the field of cancer research, and have been elegantly reviewed elsewhere [20, 31]. However, with the possible exception of the TP53-null mammary tissue transplantation model which forms multiple tumor types [32], a single GEMM typically forms a relatively homogeneous set of tumors, and thus does not recapitulate the range of human disease [33]. As a consequence, multiple GEMMs must be interrogated to show generalizability of results. Thus, for the purposes of this review, we will focus exclusively on human cells grown in vivo.

Cancer researchers have engrafted long-established human breast cancer cell lines into immunocompromised mice as experimental models for decades. However, many long-established cell lines do not grow in vivo (so far), and even if they did, many have drifted genetically over time, and can vary lab-to-lab, such that it is questionable that they accurately recapitulate the biology of the tumors-of-origin [34]. It appears likely that these changes contribute to the disappointingly abysmal failure rate of more than 90% of cell line-based results to translate into the clinic [35–37]. As a consequence, there is a strong interest in improving preclinical animal models to recapitulate human disease more faithfully and more consistently. In response to this need, quality-controlled patient-derived models have emerged as powerful preclinical/translational tools that are beginning to yield clinically actionable results, some in real time [12].

### Immunodeficient Mouse Strains

In the 1960s, the athymic and hairless “nude” mouse was the first immunocompromised strain characterized [38, 39]. Nude mouse phenotypes are the consequence of homozygosity of a spontaneous mutation in the *Foxn1*^*nu*^ gene (previously *Hfh11*^*nu*^). These mice have defective thymic development and thus lack T cells. They also show defects in B cell development, but good NK cell activity. While nude mice will tolerate the growth of many human cell lines, they do not support efficient engraftment of human tissue. Experimentally, nude mice have some advantages, particularly in imaging studies for which the presence of hair can interfere with image collection and analysis considerably.

A subsequent breakthrough was the generation of the severe combined immunodeficiency (*scid*) mouse strain. These mice lack both B and T lymphocytes due to a homozygous mutation in the gene coding for the catalytic subunit of DNA-dependent protein kinase (*Prkdc*^*scid*^) [40]. This mouse strain could be engrafted with human cells, albeit at a low rate, likely due to an intact innate immune system.

A critical improvement was the backcrossing of *scid* mice to the non-obese diabetic (NOD) strain which does not express the H-2g7 major histocompatability complex (MHC) haplotype and possesses a CTLA-4 alteration causing diabetes-induced autoimmunity [39, 41]. The resulting NOD-*scid* strain lacks T- and B cells and has lower NK-cell activity allowing a higher rate of engraftment of human hematopoietic stem cells, leukemias, as well as breast and other cancers [7, 41–43].

Two subsequent breakthroughs allowed researchers to engraft human tumors at significantly higher rates. The first was the development of the *scid*/*bg* mouse line, which combined the *scid* mutation with the beige (bg) mutation of the lysosomal trafficking regulator (*Lyst*) gene [44]. Beige mutants have, among other defects, defective cytotoxic T cells, severely compromised NK cells, as well as defective granulocytes and platelets. The *scid/bg* mouse was shown to accept human lymphoid cells with high efficiency, and has been used subsequently to host a variety of normal and malignant tissues [3, 7, 45, 46].

The second breakthrough was achieved by crossing existing immunodeficient mice with homozygous mutations targeting the interleukin-2 receptor common gamma chain (*IL2Rγ*) locus, which diminishes NK cell activity [47]. First, an *IL2Rγ* mutation, was introduced in NOD/Shi-*scid* mice generating the NOD/Shi-*scid Il2rg*^*null*^ (NOG) strain [48]. Subsequently, introduction of an *IL2Rγ* mutation to NOD/LtSz-*scid* mice generated the NOD/LtSz-*scid Il2rg*^*null*^ (NSG) strain [49, 50]. While not yet tested in a head-to-head comparison, *scid/bg* and NSG hosts yield breast cancer PDX at a comparable rate [7].

Strains have also been generated using mutations in the *Rag1* or *Rag2* genes required for V(D)J recombination in antibody production as well as recombination of T cell receptors [51–53]. *Rag1* and *Rag2* deficient mice have small lymphoid organs that do not contain mature B and T lymphocytes, as well as defective NK cells. Rag-deficient mice have been shown to have enhanced resistance to irradiation and tolerate some chemotherapies better than other immunocompromised strains [54–56]. Subsequent introduction of *Il2rg*^*null*^ genotype in conjunction with the *Rag2*-deficient strain generated the Balb/c *Rag2*^*null*^* Il2rg*^*null*^ (BRG) mouse, which also lack B and T lymphocytes as well as NK cell activity [57]. The cross of NOD-*scid IL2Rγ*^*null*^ with NOD-*Rag1*^*null*^ mice generated the NOD-*Rag1*^*null*^* IL2Rγ*^*null*^ (NRG) strain [55].

This tremendous progress in genetic manipulation has enabled researchers to engraft human cells to study hematopoiesis, the immune system, infectious disease, and cancer to greater effect.

### Humanized Mouse Models

Humanized mouse models have been developed to examine interactions between immune components and human tumors. While various human immune components have been reconstituted in mice, study of tumor immunology is more complex as the models must tolerate engraftment of both human tumor and immune cells. Currently, peripheral blood mononuclear cells (PBMCs) and human CD34^+^ hematopoietic stem cells (HSCs) are the two major immune cells that have been successfully engrafted in immunodeficient mice to establish a functional immune system. These cells have been used to develop three main models: Hu-PBL (peripheral blood lymphocytes), Hu-CD34 + , and BLT (bone marrow-liver-thymus) mice.

The simplest model, Hu-PBL, is derived by engrafting human leukocytes into immunodeficient mice [58]. In this model, there are low levels of human B and myeloid cells. However, human T cells are present and remain functional in the murine host. A major caveat of this model is the development of graft-versus-host disease (GvHD) making it suitable only for short-term studies [59, 60]. Despite this short time frame, Hu-PBL model has been used to demonstrate the ability of a CD137 antibody to inhibit cell line xenograft tumor growth [61]. This model was also used to test the use of radioactive labeled PD-1 antibodies to monitor T cell infiltration in lung cancer cell line xenografts [62]. Additionally, the Hu-PBL model was used to evaluate delivery of an adenoviral vector to modify rare cell types in a breast cancer xenograft model including circulating tumor cells, micrometastases, and CD4^+^ human T lymphocytes [63].

Hu-CD34^+^ mice are generated by isolating human CD34^+^ HSCs from peripheral blood [49], bone marrow [64], fetal liver [64], or umbilical cord blood [57], and engrafting them into irradiated immunodeficient host mice. Although all human hematopoietic lineages are present in this model, frequencies of individual cell types are highly variable mouse-to-mouse, and some are not fully functional. Most human B cells are immature because B cell differentiation is inhibited and survival in the periphery is limited, resulting in accumulation of B cell precursors in the spleen [65, 66]. Additionally, CD8^+^ T cells and NK cells display some level of functional impairment [67]. In an early application of this model, Wege and colleagues co-transplanted CD34^+^ HSCs and human breast cancer cells in NSG mice and observed tumor growth and dissemination, as well as tumor-specific T cell and NK cell activation [68]. These findings demonstrated Hu-CD34^+^ mice are a viable model for preclinical evaluation of immunotherapies and dissecting mechanisms of resistance in breast cancer. Recently, Hu-CD34^+^ mice have been used to evaluate pre-clinically response to immunotherapy. Several PDX models of various cancers, including triple-negative breast cancer (TNBC), were engrafted in Hu-CD34 + and NSG mice and treated with a PD-1 inhibitor, pembrolizumab [69]. Treatment with pembrolizumab resulted in growth inhibition in Hu-CD34 + mice, however this was dependent on the HSC donor.

Humanized bone marrow-liver-thymus (BLT) mice are generated by transplantation of human bone marrow, fetal liver, and thymus into the subrenal capsule of an adult immunodeficient mouse. Simultaneously, the mice are given an intravenous injection of CD34^+^ HSCs derived from the same fetal liver [70, 71]. The transplantation and subsequent development of a human thymus organoid leads to generation of a more robust peripheral immune system. Notably, T cells developed in the thymus organoid are capable of activation by human antigen presenting cells, leading to potent human MHC-restricted T cell responses [72]. This may allow a T cell response to human tumor engraftment, which can recapitulate the complex T cell and tumor biological interaction. However, T cells with affinity for mouse MHC are not eliminated, resulting in higher incidence of GvHD than other Hu-CD34^+^ models [73].

The Jackson Laboratory (JAX) and others have developed a portfolio of CD34^+^ and PBMC humanized mouse strains. All strains available from JAX are based on the NSG mouse, which is permissive to engraftment of human CD34^+^ and PBMCs. Presently, JAX has two developed and characterized humanized strains. Hu-NSG-SGM3 are triple transgenic mice expressing human IL3, GM-CSF (CSF2), and SCF (KITLG) in a NSG background [74]. These cytokines support the stable engraftment of human myeloid lineages and regulatory T cell populations. NSG-IL15 mice express human IL15 in an NSG background. Expression of IL15 enhances the development of human NK cells in mice engrafted with CD34^+^ cells. Hu-PBMC-NSG humanized mice are available from JAX, created by engrafting human PBMCs in NSG or NSG-SGM3 mice. The rapid engraftment rate enables short-term studies requiring mature human T cells. These models offer investigators the ability to evaluate immunotherapies using PDX models.

Though humanized mice are a powerful tool that can advance immunotherapy research, they do not fully recapitulate the human immune system, and may be cost prohibitive for some research groups. Notably, most research using humanized mice has used CDX as opposed to PDX models. It is critical to obtain matched patient PBMCs to evaluate immunotherapies in a humanized PDX model, which may not always be feasible. A major limitation regarding the use of humanized mice and PDX models to study immunotherapies is the time it takes to engraft human immune cells and PDX tissue. As a result, the window to conduct a preclinical immunotherapy trial, with an adequate time to monitor disease recurrence, may be small. Therefore, significant efforts are needed to improve the humanized models available for routine use.

### Immunodeficient Rat Strains

Although mice are commonly used for cancer research, the laboratory rat is a viable alternative that possesses distinct advantages. The larger size of the rat offers the ability to perform non-invasive imaging [75, 76], to grow tumors up to double the diameter possible in mice [77], and easy surgical manipulation. Interestingly, rat tumors more closely resemble certain aspects of human breast cancer pathology than mice. For example, whereas exceptionally few mouse tumors are ER+ , ~70% of rat mammary tumors are ER+ and are estrogen dependent, compared to ~75% ER-positivity in humans [78–81]. As a consequence, immunocompromised rats may be a more suitable host for hormone dependent breast cancer [7].

For decades, development of transgenic rat models of cancer lagged behind mice due to the absence of germline-competent rat embryonic stem (ES) cell lines. Following the successful derivation and maintenance of germline-competent rat ES cells [82, 83], rats can now be genetically modified at will.

Historically, using xenografts in rats was uncommon. Recent advances in the development of immunodeficient rats has increased their utility in studying human cancer. The nude rat (RNU) was first characterized in 1978. This model lacks T cells, like the nude mouse, but has functional B and NK cells making engraftment of human cells challenging [84]. In fact, several studies in the 1980s demonstrated the nude mouse could engraft and support human tumor cells better than the nude rat, possibly due to age-dependent changes in immune competence [85, 86]. Despite this, the human breast cancer cell line MDA-MB-231 has been successfully engrafted orthotopically in nude rats [77]. Nude rats have also been irradiated to increase engraftment success rates [87].

Many genes targeted to develop immunodeficient mice have also been exploited to develop immunodeficient rats. The *Rag1* and *Rag2* genes have been targeted to develop immunodeficient rats with decreased proportions of functional T and B cells, but these models also have elevated levels of NK cells, like their mouse counterparts [39, 88–90]. A more severely immunocompromised rat strain F344-*SCID*-γ (FSG) was developed by targeting the *Prkdc* and *Il2rg* genes (*Prkdc*^−/−^ *Il2rg*^−/^) [91]. These severely immunocompromised rats lack T, B, and NK cells. Though human stem cells, tumors, and hepatocytes could be engrafted successfully, these rats are smaller and weigh less than their wild-type littermates.

Another severely immunocompromised strain, SD-RG rats, was developed by knocking out *Rag1*, *Rag2*, and *Il2rg* [92]. SD-RG rats have severely impaired development of lymphoid organs and lack mature T, B, and NK cells. Importantly, lung cancer PDX models have been established in this model. Recently, Hera BioLabs developed a Sprague–Dawley *Rag2/Il2rg* double knockout (SRG) rat also lacking mature B cells, T cells, and NK cells. The SRG rat exhibited efficient tumor take rates with cell lines, including the difficult prostate cancer cell line VCaP and patient tissue [93].

Immunodeficient rats also have the potential to be humanized to study immunotherapy and human tumor/immune cell interaction. RRGS (*Rag1*^−/−^ *Il2rg*^−/−^) and NSGL (*SIRPα*^+^*Prdkc*^−/−^*Il2rγ*^−/−^) rats have been successfully engrafted with human PBMCs and CD34^+^ cells, respectively, to reconstitute the human immune system [80, 94].

### Immunodeficient Pig Strains

Large animal models such as pigs are advantageous for research since they are more anatomically and physiologically similar to humans than are rodents, and in many cases recapitulate human disease pathogenesis more closely [79]. For example, pigs are comparable to humans with regard to size, genetics, immunology, and metabolism [78, 95, 96]. Expense aside, these benefits could make pig models a powerful translational research tool in the future.

In the past decade, immunodeficient pigs have been established through both mutagenesis and discovery of natural mutations. The first *scid* pig, described in 2012, was able to support engraftment of pancreatic cancer and melanoma cell lines [97]. Subsequent analysis of this model revealed these *scid* pigs have two naturally occurring mutations in the *Artemis* (*DCLRE1C*) gene, which impairs V(D)J recombination [98, 99]. This leads to T and B cell deficiency, although NK cells are functional [99, 100]. Initially a melanoma cell line and a pancreatic cancer cell line were successfully engrafted into the ear tissue [97], and recently an ovarian cancer cell line was successfully transplanted into the neck and ear tissue of *scid* pigs [101]. Other human cells have been engrafted into *scid* pigs as well, including induced pluripotent stem cells and vascular grafts [102, 103]. However, breast cancer cell lines have not yet been engrafted into an immunodeficient pig.

Another model was developed in 2012 by mutating the *IL2Rγ* gene, which leads to defective T and NK cells [104]. Other groups have targeted the *RAG1* and *RAG2* genes to generate *scid* pigs lacking B and T cells [104, 105]. In 2016, a *RAG2*/*IL2Rγ* double knockout was developed, which lacked B, T, and NK cells [106]. Introduction of mutant *IL2Rγ* into an Artemis null background also eliminated B, T, and NK cells [107]. This model was successfully engrafted with human CD34^+^ cells, which resulted in circulating human T cells and human leukocytes in lymphoid organs [108]. Taken together, this model represents a significant step forward in the development of humanized pig models.

*Scid* pigs require special housing to maintain viability in research settings. In conventional settings, *scid* pigs succumb to disease between 6 and 12 weeks of age [109]. Biocontainment facilities have been designed at Iowa State University to house *Artemis*^−/−^ *scid* pigs and limit micro-organism exposure [110]. Additionally, small isolators have been developed to deliver and rear *IL2Rγ* mutant *scid* pigs. This protocol was able to maintain germ-free *scid* pigs for a period of 12 weeks, which would allow longer-term experiments [111].

### Immunodeficient Zebrafish

Though zebrafish (*Danio rerio*) are an established model to study toxicology and development, they are increasingly used to study human cancer. Due to their small size, rapid ex vivo fertilization and development, genetic tractability, inexpensive housing costs, and ease of conducting large scale screens, zebrafish possess distinct advantages as a model system [112].

Zebrafish embryos and larvae are particularly well-suited for xenotransplantation because their adaptive immune system begins developing around 7 days post fertilization and does not fully develop until two to four weeks post fertilization [113]. In a pioneering experiment by Lee et al. in 2005, human melanoma cell lines were engrafted into zebrafish embryos [114]. No tumors formed, but the cells migrated throughout the embryo and retained their de-differentiated phenotype. Many cell lines have been engrafted successfully into zebrafish embryos, including leukemia [115], ovarian cancer [116], pancreatic cancer [117, 118], glioma [119], and breast cancer [120]. The yolk-sac is the preferred transplantation site, but researchers have also used the caudal vein, perivitelline space, pericardial cavity, and hindbrain ventricle [121]. Cell line engraftment approaches have been used to investigate cancer stem cell self-renewal [122], tumor angiogenesis [123], as well as invasion and metastasis [120, 124]. Zebrafish larvae xenograft models are a powerful tool for high throughput drug screening. A recently developed methodology, ZeOncoTest, was validated by treating multiple cell lines with known effective drugs [125]. The results recapitulated growth and invasiveness for all tested tumor cells as well as the expected efficacy of the compounds.

Despite their efficacy for drug screening, metastasis, and angiogenesis, zebrafish larvae have notable limitations. In drug studies, larvae are treated by adding drugs directly to the water, which makes accurate assessment of dosing, PK, and PD difficult and requires more drug to accommodate the volume. Engrafted zebrafish larvae are raised at non-physiological temperatures <34 °C resulting in altered proliferation rates. Also, engrafted larvae do not develop histologically similar tumors as compared to humans and these studies are limited to the first weeks of life, before the fish develop an immune system.

To address these limitations, a variety of immunocompromised zebrafish strains have been created using *rag1* [126], *rag2* [127], and *prkdc* [128] mutants. In a major step forward, the Langenau group generated a *prkdc*^−/−^, *ilrga*^−/−^ *casper* strain of zebrafish [129]. This model lacks both adaptive and NK immune cells, and allow engraftment of a variety of human cancer cells at 37 °C. Importantly, patient-derived cells were successfully engrafted from several different tumor types, including breast cancer. Preclinical assessment of Olaparib (PARP inhibitor) and temozolomide (DNA-damaging agent) confirmed the anti-tumor responses observed in mouse PDXs, with similar pharmacokinetics. Recently, the first humanized zebrafish was generated, which expresses human-specific cytokines [130].

Drug administration and dosing in adult zebrafish are important considerations for translational studies. Adult zebrafish have been administered drugs by intraperitoneal injection [131, 132] and oral gavage [129, 131]. To date, empirical testing has largely been used to determine dose-conversion factors. Additional study is required to optimize drug dosing and conversion factors between zebrafish, mice, and humans. Drug response in zebrafish models has been measured in several ways: direct imaging of tumor cells [131], fluorescent imaging of tumor size (by transducing cancer cells with a fluorescent reporter) or with FUCCI to visualize cell cycle phases [129], ultrasonography [133], and measure of cell numbers [134] and tumor surface area [129].

Despite being a newer model, zebrafish offer a unique approach with advantages over other models in terms of scale, cost, and speed of model development. An ongoing clinical trial (NCT03668418) aims to assess the predictive power of zebrafish larvae PDXs. If high predictive power can be established, the low cost and high throughput drug screening capabilities may render the zebrafish model an attractive alternative for precision cancer therapy.

### The Embryonated Chicken Egg Cam Model

**Fig. 1:**
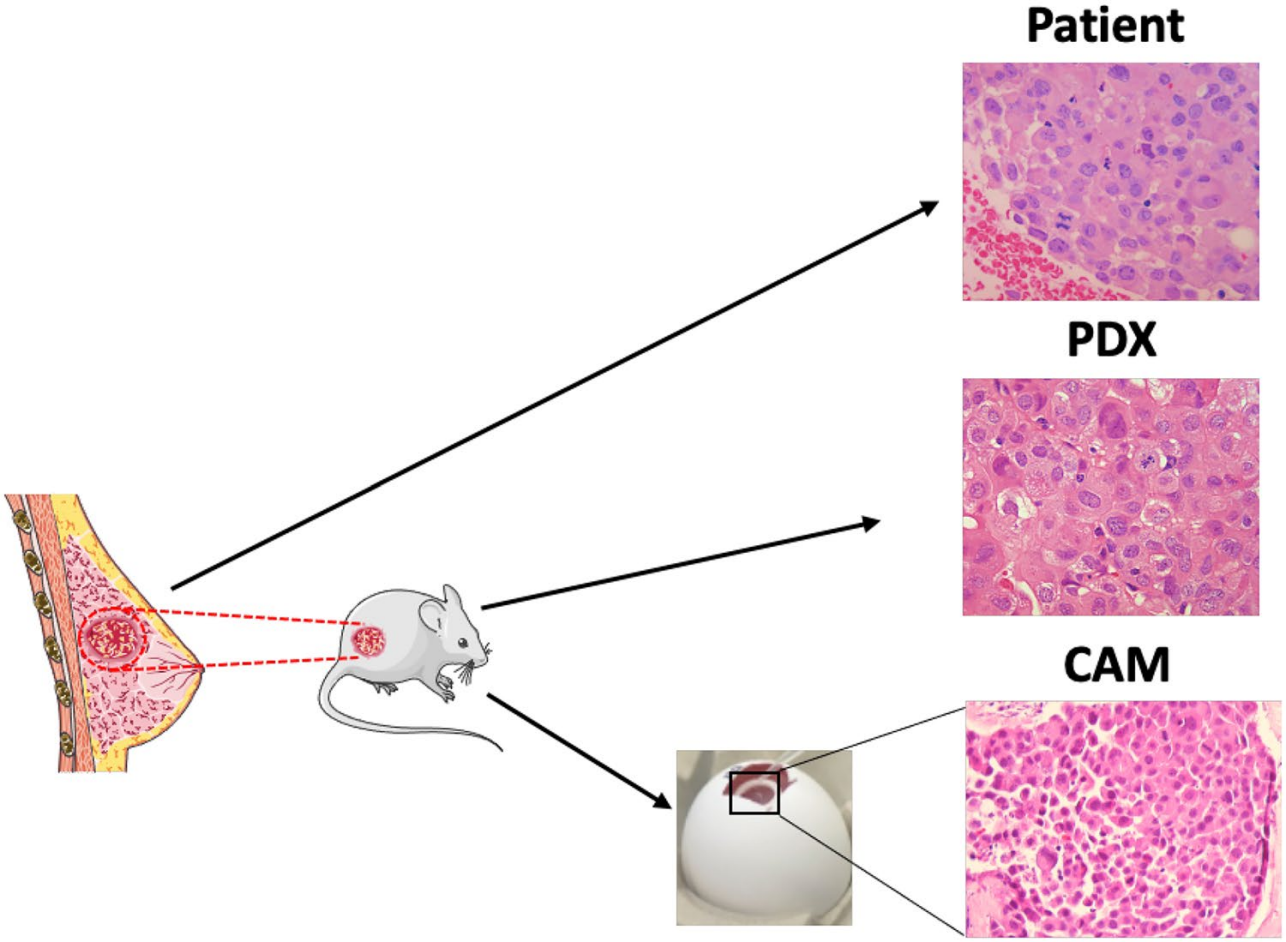
Breast PDX transplantation workflow. Patient-derived breast tumors are collected and engrafted on immune-compromised mice for PDX establishment. Established PDX can be tested on mice or can be transplanted on to the CAM. Hematoxylin and eosin stained tissues from a triple-negative PDX model on the original patient tissue, mouse, and CAM are represented

The physiological functions of the chick embryo chorioallantoic membrane (CAM) include serving as a gas exchange nexus, calcium mobilization vessel, and transporting sodium and chloride ions out of the waste-storing allantoic cavity [135]. The vascular network interlaced within the CAM supports and nourishes the developing embryo and can likewise support patient-derived xenograft tumors and cancer cell lines (Fig. 1). In addition to its vasculature-rich environment and accessibility, the CAM is naturally immune-deficient during most of the embryogenesis phase. These characteristics allow for growth of cell lines as 3-D organoids or primary human tumor tissue until a mature adaptive immune system rejects acutely-growing xenografts. The realization of the CAM as a self-contained in vivo model for cancer research has yielded various tumor models [136, 137]. The highly vascular CAM has also been used extensively as an in vivo tool to study effects of angiogenic factors and biomaterials primarily due to the ability to continuously monitor vessel changes [138–141].

Cell and primary tumor xenografts on the CAM form three-dimensional, neovascularized tumors, and maintain properties of in vivo cancer cells often lost in two-dimensional or rudimentary three-dimensional tissue culture models [142]. Examples of properties lost include: an adequate tumor microenvironment, tumor angiogenic properties, and complex cell to cell interactions. These features make the CAM xenograft models ideal for studying biological processes such as cell growth, invasion, angiogenesis, and metastasis of human tumor cells into the developing chick embryo within a two-week period [142, 143].

The recognition that the chicken egg CAM could be used as a human PDX scaffold came from early studies performed by Holland Stevenson in 1918 and Hurst in 1939, where several patient carcinomas that were predominantly from breast were engrafted on the CAM [144, 145]. Results from the Stevenson studies indicated that CAM-engrafted human tumors do not propagate well with the basic nutrients provided by the CAM, although they did resemble the parent tumor [145]. Hurst et al. would repeat the experiments on the CAM using carcinomas from various different patients including breast tumors and were able to generate viable tumor xenografts on the chick embryo scaffold [144]. The success of engraftment continued to improve modestly with Sommers in 1952 and Kaufman in 1956, who both attempted to engraft breast carcinomas amongst other tumor [146, 147]. Surprisingly, not much progress has been made since then in breast cancer modeling using the CAM-PDX platform. Nonetheless, there has been increasing interest in credentialing the CAM model as a PDX-sustaining avatar using tumors derived from multiple sites [13, 148–154].

## In Vivo Models of Human Cancer: Established Cell Lines vs. Patient-Derived Tissue

Over the last several decades, a large number of cell lines were generated from cancers of all types. Many of these cell lines are now curated into publicly available sets such as various American Type Culture Collection (ATCC) panels [155, 156] and panels specific for breast cancer, including the SUM cell line panel [157] (https://sumlineknowledgebase.com), as well as a set of 51 cell lines (originally, now 45) [158] that partially overlap with the ATCC and SUM collections. Individual cell lines from these collections have proven instrumental for defining molecular mechanisms underlying cell behaviors, and as collections, there are data suggesting relevance for prediction of drug responses in patients [159–161].

Recently, omic and drug response data from 947 cancer cell lines were compiled into a Cancer Cell Line Encyclopedia with the goal of identifying candidate drug targets [162, 163]. Similar cell line-based studies using combined omic analysis and drug response have been conducted to identify molecular correlates of drug response including the Genomics of Drug Sensitivity in Cancer (GDSC) project (www.cancerRxgene.org) [164–166], and the Cancer Target Discovery and Development (CTD2) Project [167] (Cancer Target Discovery and Development (CTD2) Data Portal (ctd2.nci.nih.gov/dataPortal/). In aggregate, these data are now being used to, among other things, attempt to predict drug response for personalized medicine using a variety of computational methods [168–172].

The three above mentioned studies were conducted in vitro in 2-dimensional culture conditions, and while some results could be translated clinically, these and similar studies have arguably yielded less predictive insight than one might hope. The reasons for this may stem from observations from a number of studies that showed that gene expression patterns and drug responses using the same cell line grown in 2-dimensional vs. 3-dimensional culture are different [173], and it would stand to reason that if grown in vivo their patterns would be different still. Thus, the ability to generate clinically relevant data using cell lines may be limited unless grown in vivo, preferably at the orthotopic site. However, this requirement would eliminate their other advantages and thus reduce their utility considerably. Further, only a subset of the established cell lines are capable of growth in the mammary fat pad of immunocompromised mice. Though this limitation can be overcome by varying the transplantation method and anatomical site used. For example, Sflomos and colleagues demonstrated classical breast cancer cell lines, including ER + lines, grow successfully in the mouse using intraductal injection [174].

CDX suffer other limitations as well. While CDXs are useful tools due to their availability, low cost, and high take rates, there are significant limitations. Cell lines are passaged numerous times in vitro prior to engraftment, which results in clonal selection and loss of tumor heterogeneity [160, 161]. Notably, different samples of the same cell line can have dramatically different gene expression patterns. An analysis of a frequently used breast cancer cell line (MCF-7) revealed a parental cell line and its three subclones displayed remarkable differences at the genomic and gene expression levels [175]. As a result, different strains of the same cell line may have differing responses to anti-cancer drugs. A drug response analysis of 27 MCF-7 strains revealed considerably different responses, with some strains responding completely to the tested compounds, while other strains were non-responsive [176]. Additionally, some studies have demonstrated CDXs have poor predictive value of response to therapeutics. A comparison of findings from 39 compounds tested in both CDX models and Phase II clinical trials at the National Cancer Institute’s Developmental Therapeutics Program found no close correlation, casting doubt on the relationship between results in CDX pre-clinical models and clinical trials [177]. In fact, transcriptomic comparison of clinical samples to established cancer cell lines revealed all cell lines bear more resemblance to each other rather than the clinical samples they are intended to model [161].

Indeed, a comparison of molecular features of 68 breast cancer cell lines to 1375 breast tumors in TCGA showed that while there were residual similarities between the cohort of cell lines vs. the cohort of breast tumors, there were significant differences in mutation rates and genomic copy number alterations, with cell lines higher in both categories, likely due to the accumulation of genomic alterations as a function of handling conditions and passage over time [178]. This said, direct comparison of bulk RNA gene expression in cell lines (100% epithelial) vs. bulk human tumor (mixed epithelium and stroma) may account for some of this analytical difficulty in that the admixture of epithelial and stromal gene expression may not be comparable to epithelium only gene expression.

To complement long-established cell lines and to help overcome at least some of the perceived limitations, a number of groups began to develop collections of Patient-derived Xenografts (PDX) from a variety of organ sites in addition to breast (please see the PDMR—https://pdmr.cancer.gov/, the BCM PDX Portal—https://pdxportal.research.bcm.edu/, EuroPDX—https://www.europdx.eu/), and the Seven Bridges PDXNet Portal (https://portal.pdxnetwork.org/). With the cancelation of the NCI-60 cell line panel for use in drug screening by the NCI [179], the use of PDX models quickly became the standard platform for preclinical and co-clinical trial testing. Since PDX models are established directly from patient tissue, they retain 3-dimensional architecture and signaling. Although patient stromal cells are quickly replaced by murine stromal components, fidelity of the cancer is retained when evaluated by histological, genomic, transcriptomic, and proteomic methods. PDX models also recapitulate the patient tumor response to therapeutic agents making them a valuable tool for precision oncology [180–183].

## In Vivo Model Credentialing

### Annotation

A critical component for all PDX collections is the availability of high quality clinical and molecular annotations for the patients yielding PDX. Such annotations can be extremely useful for PDX model choice in drug studies if the patient tumor was treated with a similar agent and can also be used analytically when evaluating drug responses and other PDX phenotypes.

To this end, the two major PDX consortia, the NCI PDXNet in the United States and EuroPDX in Europe, have made a concerted effort to agree upon the minimal information (MI) that should be abstracted in a de-identified manner from the clinical records by qualified staff, and associated with the PDX model as part of the credentialing effort [14]. MI includes tumor origin (e.g. breast) clinical setting (neoadjuvant, adjuvant, metastatic), age at collection, pathological diagnosis (with H&E staining), tumor grade and stage, clinically relevant biomarker expression (e.g. estrogen and progesterone receptors (ER/PR) and ErbB2 (HER2) amplification and/or overexpression, as well as BRCA1/2 germline mutation status in the case of breast cancer), therapeutic treatments and associated responses, metastatic sites in the patient, as well as patient demographic information including age at diagnosis, race, ethnicity, and vital status.

To these patient-centric data, PDX-centric data are annotated as well, including transplant conditions under which the model was generated (e.g. host, tissue state at transplant (fresh or viably frozen), matrigel or not, and any supplements that may be required such as estradiol pellets or estradiol-containing drinking water), as well as matching immunohistological imaging by which to compare to the tumor of origin. To these data are added multiple “omic” data types including whole genome and whole exome DNA sequencing (WGS/WES) (mutations and copy number variation), RNAseq transcriptomics (human and mouse), and mass spectrometry-based proteomics (human and mouse). In order to provide the most robust cohort of patient-PDX matched data, it is optimal to obtain germline patient specimens from either blood or normal tissue and patient tumor tissue from the sample which created the PDX model (source tissue). Germline samples are analyzed by the appropriate omic analysis methods and used to evaluate the fidelity of the PDX model.

Collection of MI and “omic” data, even on a small number of patient samples and PDX models in a collection, is a major undertaking and requires close coordination with clinical staff who enter information into the clinical record, research coordinators who conduct the abstracting, tumor bank personnel who register, store, and distribute the tissue, and research laboratory staff who generate the PDX and associated data. Once collected, however, the vast amount of patient and PDX related data lead to management, analysis, and display challenges.

To deal with such challenges, several groups are developing database infrastructure and software to support PDX-based work. At BCM, we are using two complementary software tools and their underlying database infrastructure to accomplish sample and data management. These are OpenSpecimen [184], which allows tracking of samples and some basic, specimen related, annotation, as well as Acquire [185], which allows full clinical and PDX model annotation on a cohort basis. The data collected and stored in these two databases is then integrated into a web-based PDX Portal (https://pdxportal.research.bcm.edu/) that is used both for PDX collection management (e.g. What lines do we have? Which are public and which are held privately? What data do we have on those lines? What is missing? etc.), as well as for data analysis and display (e.g. What PDX lines express a gene/protein of interest at high/low levels? What lines have mutations or copy number alterations in a gene of interest?). The data analysis and display functions are useful for selecting models for studies, particularly drug studies, where knowing that a candidate target is expressed, and at what levels, can be informative for anticipating outcome of the treatment [186–188].

The ultimate goal of the BCM PDX Portal is to allow PDX generators to manage their collections independently and efficiently, and to allow PDX users easy, real time, access to PDX-related annotation and omic data. To this end, the PDX Portal is able to host data from any other institution, should they so desire. Currently the PDX Portal contains public data for multiple cancer types from BCM, the Huntsman Cancer Institute, Texas Children’s Hospital, and the University of Basel.

While the information in the BCM PDX Portal is already compatible with the PDXFinder, PDXNet, and PDMR portals, in future efforts, PDX data held at BCM may be integrated automatically with these web-based resources.

## PDX Model Quality Control

### Initial QC

Once PDX models are established and determined to be stably growing (tumor formation at transplant generation three), several quality control measures need to be taken to ensure the new models are biologically relevant. For breast cancer models, immunohistochemical staining is necessary to compare the PDX tumor’s histology and biomarker status to the patient tumor of origin. Hematoxylin and eosin (H&E) staining is used to verify the histology of the PDX tumor and to evaluate the size and location of necrotic areas. Also, human and mouse cells can be distinguished using staining with a human-specific pan cytokeratin antibody or staining for Alu elements [29]. CK19 can be added as an additional epithelial marker and is often used clinically in panels to determine the aggressive nature of breast tumors [189–191] but some models are negative for this marker. Staining for ER, PR, and HER2 expression is done to evaluate the retention of breast cancer biomarkers in the PDX model. To verify overexpression of the ERBB2 gene, FISH testing is performed on models showing equivalent or positive staining by IHC. Ki67 is also included in the initial IHC panel to evaluate the percentage of cells that are dividing.

Ensuring the safety of laboratory personnel and mouse colonies is of utmost importance in all PDX programs. After a model is deemed stable, pathogen testing for common human viruses, murine viruses, and bacteria by qPCR should be completed. The size and scope of these panels may vary depending on institutional requirements, but human virus testing should include, at a minimum, human immunodeficiency virus (HIV1/2), hepatitis virus A (HepA), hepatitis virus B (HepB), hepatitis virus C (HepC), and Epstein-Barr virus (EBV). Due to the severe immunodeficient status of mice used for PDX establishment, bacterial testing of PDX models should include mycoplasma species, corynebacterium species, and, specifically, *Corynebacterium bovis (C. bovis)*. *C. bovis* is a common environmental contaminant which causes “scaly skin disease” in immunodeficient animals and can ultimately interfere with research activities by inhibiting tumor take rate. Routine monitoring is vital as infection can quickly spread through a colony. Infected animals must be removed and the model must be re-derived from uninfected stock. A comprehensive mouse virus panel must also be completed and should include lactate dehydrogenase-elevating virus (LDEV), which has been previously known to contaminate common reagents with animal components like matrigel.

Xenograft-associated lymphoproliferative disease (XALD) is caused by the proliferation of atypical lymphocytes after implantation which may outgrow the tumor tissue. The vast majority of these cases originate in human tumors that are EBV positive, which is why inclusion of EBV in the human pathogen testing panel is critical. Immunohistochemical staining is used to determine if a lymphoid tumor has developed and can determine the lineage. Antibodies specific for human and mouse CD45, a pan-leukocyte marker, can confirm if a lymphoid outgrowth is of human or murine origin. If histology and CD45 staining indicate that a PDX is possibly an XALD, CD20 and CD3 can be used to distinguish between human B cells (CD20 +) and T cells (CD3 +) [192]. Many of these lymphoproliferative outgrowths are very similar to diffuse large B cell lymphoma (DLBCL) and are classified as XABLD. These models are positive for EBV, CD45, CD20 and a large majority stain positive for PD-L1 [193]. Transcriptomic profiling clusters these models with DLBCL. Several groups have shown that treatment with rituximab at the time of implantation greatly reduces the number of XALD outgrowths by depleting the CD20 cell population [192, 194, 195].

To validate that the PDX model is derived from the correct patient of origin, short tandem repeat (STR) profiling should be performed on DNA from the PDX tumor tissue and either a germline sample or tumor tissue sample from the patient. STR fingerprinting should be done every five transplant generations as a quality control measure to confirm the identity of the model. This is especially important when laboratories have multiple PDX models growing at the same time. When tumor treatment studies are performed and published, tissue from a control mouse should be STR tested as confirmation that the results are from the correct model.

### Omics QC

In addition to biomarker expression comparison and confirmation of genomic relationship with the patient and tumor of origin, it is preferable that PDX be compared to their tumor of origin using any number of molecular ‘omics’ platforms at the DNA, RNA, and protein levels among other possibilities, the choice of which depends on the questions being asked experimentally.

In the field in general, a minimal omics characterization at the DNA level would include either targeted or whole exome sequencing of the PDX, with matching tumor of origin and patient germline whenever possible. From these data, mutations can be identified using either a “tumor only” bioinformatics platform, or in direct comparison with the patient germline sequence. In addition, genomic copy number variation and variant allele frequencies can be calculated.

At the RNA level, early studies made use of gene expression arrays, which demonstrated consistency of gene expression patterns between PDX and matching primary tumor, in most cases, as well as excellent stability of gene expression across transplant generations [3, 7, 13, 14, 16–19, 22–27, 76]. More recent studies make use of RNAseq technology. Here again, it is preferable to obtain data from both the PDX and the primary tumor from which it was derived for direct comparison of the fidelity of gene expression patterns between the two. However, generalized comparison of RNAseq gene expression patterns between PDX and patient tumors show remarkable consistency [15–17, 19].

As with gene expression arrays, RNAseq data comparisons demonstrate that gene expression patterns in PDX are remarkably consistent with those of primary tumors [15–17, 196]. Recently, PDXNet has worked to standardize analysis methods and benchmark them against simulated data [15–17]. However, the fact that stromal cells present in bulk PDX are mouse rather than human does pose analytical challenges when trying to compare bulk PDX expression patterns with primary tumor expression patterns. This major difference must be taken into account when attempting to subtype PDX tumors relative to their tumor of origin. For breast cancer, the PAM50 classifier performs reasonably well for this broad purpose [197].

With respect to protein expression, some work has been done in this area using Reverse Phase Protein Array technology, which, as with RNA-based gene expression arrays, showed remarkable stability of protein expression, including phosphoprotein expression, up to 15 transplant generations in mice [3]. Within the last few years, mass spectrometry techniques have improved dramatically, with the ability to quantify not only the total unmodified proteome, but also phosphoproteome, acetylome, and other post-translational modifications. Human vs. mouse peptide origin can be discerned using differences in peptide mass based on amino acid composition using computational tools such as gpGrouper, which was designed for this purpose [198]. Patterns of protein expression in PDX compare favorably with breast cancers characterized by the CPTAC program [19].

### Phenotypic QC

In addition to histological comparison of the PDX with the tumor-of-origin, a few groups have reported on the metastatic behavior of PDX from the orthotopic transplantation site, particularly in relation to the observed metastasis patterns of the corresponding patient, which are largely recapitulated [3, 199–201]. Primary sites of breast PDX metastasis are lung, bone, and brain [202, 203]. In addition, PDX have also been shown to have circulating tumor cells [200, 204, 205].

## Harmonization of Clinical and Preclinical Drug Evaluation

Since most PDX models have been credentialed, and maintain fidelity with the human tumor-of-origin, they have the potential to be highly useful in translational biology. One of the main hurdles of cancer treatment is determining which therapeutic agents should progress from the bench to the bedside. The success rate of a pharmaceutical agent to make it from Phase I trials to commercial launch is <10% [206]. PDX models have the potential to improve this metric by being used as a pre-clinical therapeutic agent screening tool. Since breast cancer PDX model collections represent a wide range of “subtypes” (ER+ , HER2+ , TNBC subgroups) drugs can be screened through these models to determine what subset of patients might benefit most from a therapeutic agent. By using this approach, patient selection for a particular agent can be streamlined and possibly lead to a higher rate of drug approval. Also, as opposed to human trials where a single patient can only receive one course of treatment, mouse pre-clinical trials can be conducted where multiple treatment regimens are tested in each PDX model to determine which is the most efficacious. Novel therapeutic agents can be tested either alone, or in combination with standard of care treatments.

### Targeted vs. Screening Approaches

There are two main approaches for structuring treatment studies when using PDX models. In the first approach, existing omics data can be mined to identify a selection of PDX models that express high and low levels of the target of the drug (targeted approach); the second approach is to use a larger number of PDX models [20–30] as a “pre-clinical cohort” similar in size to some Phase I/II clinical trials (screening approach).

**Fig. 2:**
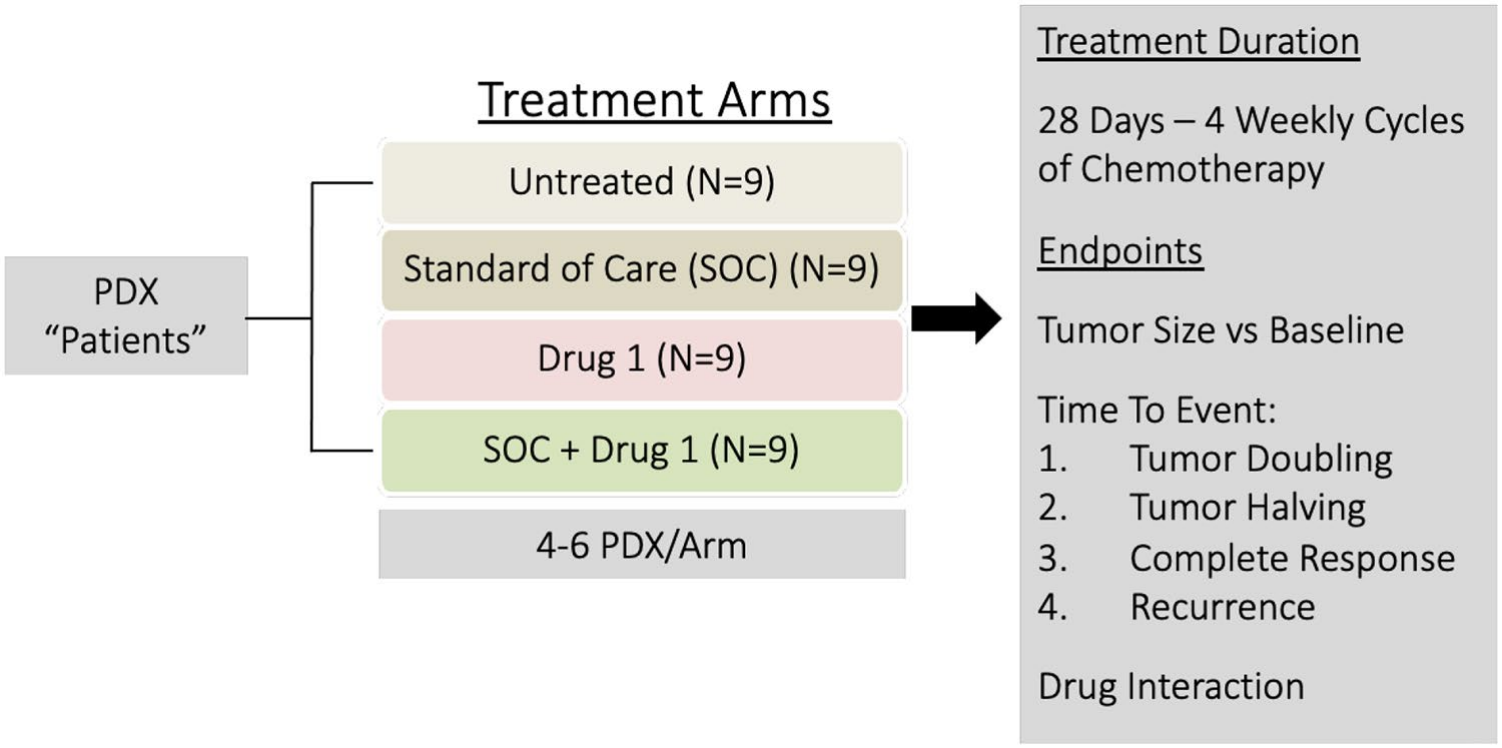
Targeted selection of PDX models. PDX “patient” models are selected with 3–4 models expressing the target alongside 1–2 negative control models that do not express the target. PDX models are divided into 4 arms: untreated, standard of care, targeted drug, and standard of care plus targeted drug. Following treatment, treatment effectiveness is evaluated and molecular assays can be performed

When using a targeted approach, 3–4 PDX are chosen based on high expression of the target and are thus predicted to respond. These are used in conjunction with at least two models that show low target expression, or lack the target entirely, and are predicted to be non-responders (negative controls) (Fig. 2). In order to obtain statistical power in a targeted approach, 9–10 mice per treatment arm are needed as a consequence of the small number of PDX used.

A limitation of the targeted approach is that only one drug is typically evaluated at a time since models are preselected based on expression of the marker of interest. A second limitation is that the drug may have activity a wider range of breast cancer subtypes than are evaluated (typically one subtype).

**Fig. 3:**
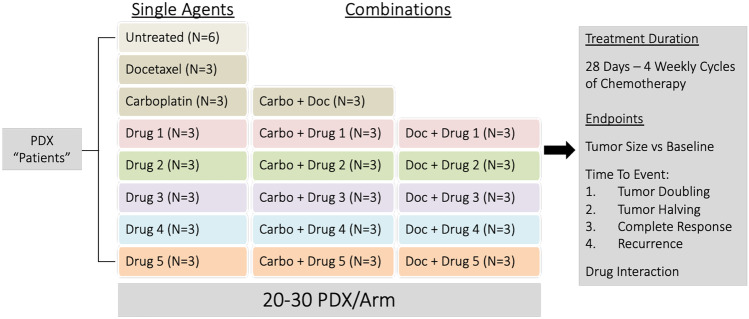
Multi-drug screening preclinical trial. PDX “patient” models are selected and divided into multiple arms including untreated, single agent, and combination treatments. Following treatment, treatment effectiveness is evaluated and molecular assays can be performed

Using the screening approach, studies are more labor intensive, time consuming, and expensive, but will inform one of a drug’s full therapeutic potential. For this study design, 20–30 PDX models are chosen either randomly, or rationally based on the drugs being used. PDX can be treated with multiple therapeutic agents simultaneously, either alone or in combinations (e.g. with standard of care agents) (Fig. 3). Because statistical power is obtained across all of the PDX tested, only six mice are required the control arm to establish the normal range of growth, while only three mice are required in each treatment arm because the desired effect size (shrinkage or complete regression) is large. A study of this size may take 1.5–2 years to complete with a highly skilled set of experimentalists. In a recently completed proof-of-concept study using 14–20 PDX models with 12–16 treatment arms, random selection of PDX proved to be inefficient for detection of responders to seven targeted agents, alone or in combination with carboplatin. Rational selection of a cohort of PDX is likely more efficient based on our experience with the targeted approach.

The advantages to embarking on such a substantial effort are numerous. First, multiple drugs can be evaluated at the same time while using fewer mice than if evaluated independently. Second, it is conceivable to evaluate drug efficacy across breast cancer subtypes. Third, the large sample size is more convincing when obtaining Institutional Review Board approval to move forward with a clinical trial. Finally, the large sample size may allow identification of molecular correlates to response and resistance.

A different approach to a screening study was taken by Novartis by performing a ‘one animal per model per treatment’ (1 × 1 × 1) preclinical trial. In this study format, 62 treatment groups (single agent or combination treatments) were tested in 277 PDX models representing 6 cancer types. They then compared the patient tumor Response Evaluation Criteria In Solid Tumors (RECIST) responses to the PDX tumor “modified” RECIST responses. Even using just one animal per study, they were able to obtain equivalent population responses in the PDX as are seen in patient cohorts [207].

## Concluding Remarks

Our ability to model human cancer in animal systems has improved dramatically over the past decade and will likely continue to evolve as existing modeling systems are refined and newer modeling systems are utilized more broadly. Investigators need to evaluate the advantages and disadvantages of each modeling system when designing in vivo experiments (Table 3).

**Table 3:** Comparison of In Vivo PDX Modeling Platforms

**Model**	**Mice**	**Rats**	**Pig**	**Zebrafish**	**CAM**
Advantages	Numerous immunodeficient strains Inexpensive Ease of genetic manipulation	Easier to surgically manipulate Larger size Develop ER^+^ tumors Produce estrogen	Anatomically similar to humans Closely resemble human disease pathogenesis Comparable metabolism to humans	Inexpensive to house, easy to breed Transparent zebrafish lines for easy visualization Can rapidly conduct high throughput drug screens Can rapidly assess chemotherapeutic sensitivity	Very permissive for engraftment Inherently immunodeficient until E18 Rich vasculature, ideal model to study angiogenesis
Disadvantages	Low engraftment rate of ER^+^, HER2^+^ breast tumors Metabolically differ from humans	Fewer immunocompromised strains Unknown utility as a host for PDX models	Expensive Neck and ear have been the only engraftment sites used Challenging for long-term therapeutic studies	Very different anatomically from humans Do not form histologically similar tumors to humans Engraftment in larvae is restricted to first few weeks of life	Inability to passage stable PDX lines over multiple transplant generations CAM-PDX histology and gene expression relative to patient tumors not well characterized

While mice have been used extensively compared to other animals, more head-to-head comparisons of immunocompromised mice are needed to evaluate the differences in PDX take rates, metastasis, treatment response, and tolerance. Advances in mouse modeling, such as the development of humanized mouse models, is a promising step toward generating a microenvironment that more faithfully recapitulates the native tumor microenvironment. Further advances in the use of alternative hosts such as the rat, pig, zebrafish, and the chicken egg CAM model may allow for novel experiments to be performed that are impractical or impossible in mice.

As these modeling platforms are used more extensively over the next several years, it will be critical to perform head-to-head comparisons to determine which platform is best suited to specific questions or techniques.

With the development and standardization of experimental techniques, generation of multiple omics datasets for each PDX collection, development and benchmarking of omic analysis pipelines, standardization of annotation, and refined pre-clinical trial design and implementation, PDXs seem poised to make major contributions to drug development and translational breast cancer research to improve patient outcomes.
