# Correction to: In Vivo Modeling of Human Breast Cancer Using Cell Line and Patient-Derived Xenografts

**DOI:** 10.1007/s10911-022-09524-8

**Published:** 2022-07-26

**Authors:** Eric P. Souto, Lacey E. Dobrolecki, Hugo Villanueva, Andrew G. Sikora, Michael T. Lewis

**Affiliations:** 1 Lester and Sue Smith Breast Center, Baylor College of Medicine, Houston, TX 77030 USA; 2 Otolaryngology-Head and Neck Surgery, Baylor College of Medicine, Houston, TX 77030 USA; 3 Department of Head and Neck Surgery, Division of Surgery, University of Texas MD Anderson Cancer Center, Houston, TX 77030 USA; 4 Departments of Molecular and Cellular Biology and Radiology, Baylor College of Medicine, Houston, TX 77030 USA; 5 Dan L Duncan Comprehensive Cancer Center, Baylor College of Medicine, Houston, TX 77030 USA; 6 Baylor College of Medicine, One Baylor Plaza, BCM-600; Room N1210, Houston, TX 77030 USA

## Correction to: Journal of Mammary Gland Biology and Neoplasia https://doi.org/10.1007/s10911-022-09520-y

The author would like to add their grant in the Acknowledgment.

The original article has been corrected.

